# SPARC Knockdown Reduces Glutamate-Induced HT22 Hippocampal Nerve Cell Damage by Regulating Autophagy

**DOI:** 10.3389/fnins.2020.581441

**Published:** 2021-01-26

**Authors:** Shuang Chen, Qin Zou, Qiang Guo, Yongmin Chen, Xi Kuang, Yukang Zhang, Yan Liu, Wengang Wu, Ge Li, Linzhi Tu, Jingyi Tong, Songrong Li, Lin Ma, Qifu Li

**Affiliations:** ^1^Department of Neurology, The First Affiliated Hospital of Hainan Medical University, Haikou, China; ^2^Key Laboratory of Brain Science Research & Transformation in Tropical Environment of Hainan Province, Hainan Medical University, Haikou, China; ^3^Epilepsy Center, Guangdong Sanjiu Brain Hospital, Guangzhou, China; ^4^Hainan Health Vocational College, Haikou, China

**Keywords:** SPARC, autophagy, glutamate, HT22, epilepsy, neurological diseases

## Abstract

Secreted protein acidic and rich in cysteine (SPARC) is a matricellular protein involved in the extracellular matrix and interactions between cells during neural development of the central nervous system (CNS). Oxidative glutamate toxicity is involved in CNS diseases, including epilepsy, Alzheimer’s disease, and ischemic stroke. However, the molecular mechanism of nerve injury is not fully understood in CNS diseases. Herein, the glutamate-induced nerve damage model was used to explore the molecular mechanisms affecting nerve damage. The levels of SPARC and autophagy were increased in glutamate-induced HT22 hippocampal nerve injury. In summary, the current study confirmed that SPARC regulates autophagy in HT22 hippocampal nerve cells, and its knockdown reduces the glutamate-induced HT22 hippocampal nerve injury by inhibiting autophagy. These findings suggested that SPARC plays a crucial role in nerve injury of CNS diseases.

## Introduction

Secreted protein acidic and rich in cysteine (SPARC) is a matricellular protein (MCP) involved in the extracellular matrix (ECM) and interactions between cells by the regulation of growth factor signaling, cytokine signaling, and cell adhesion, proliferation, and metastasis (Bradshaw, 2012). Moreover, SPARCL-1 (SC-1), also known as Hevin, is one of the SPARC family proteins involved in the migration of neurons and the formation of synapses during neural development of the central nervous system (CNS; Jones and Bouvier, 2014). A recent study showed that SPARC and SPARCL-1 are strongly implicated in the regulation of neural factors and excitatory synaptic receptors in the brain (Jones et al., 2011; Gan and Südhof, 2019; Okura et al., 2019). SPARC modulates the recovery of nerve function after CNS injury and the response of microglial cells to CNS injury (Lloyd-Burton et al., 2013). The network data also showed that SPARC is associated with Alzheimer’s disease (AD), Parkinson’s disease (PD), Huntington’s disease, and neurodegeneration with brain iron accumulation (NBIA) disease (Kumar et al., 2019). Moreover, in the AD brain, highly expressed SPARC collocates to Aβ protein deposits and contributes to cerebral inflammation and tissue repair (Strunz et al., 2019). In SPARC family proteins, the upregulated level of SPARCL-1 may be involved in synaptic remodeling of neuronal degeneration caused by epileptic seizures, and SPARCL-1 may be associated with the stress after nerve injury in the brain (Lively and Brown, 2008a). In addition, SPARCL-1 is also closely associated with reactive gliosis after transient ischemic stroke in the brain of rats (Lively et al., 2011). SPARC has attracted considerable attention as a potential chemical sensitizer to enhance apoptosis cascades (Rahman et al., 2011). Moreover, *SPARC* gene deletion reduces toxic liver injury and oxidative stress response and enhances cell proliferation (Naczki et al., 2018). However, the mechanism underlying SPARC-induced neuronal dysfunction in neuronal cells has not yet been reported.

Autophagy is an intracellular catabolism process that maintains normal cell metabolism by degrading impaired organelles and dysfunctional proteins (Shin, 2020). Autophagy also removes damaged organelles and abnormal proteins, which is conducive to the survival of cells. On the other hand, excessive autophagy can cause cell death by damaging the normal organelles (Mariño et al., 2014). Some studies have shown that SPARC is involved in regulating autophagy under various physiological or pathological conditions. SPARC activates autophagy, thereby increasing the level of cathepsin B, which in turn leads to mitochondria-mediated apoptosis (Bhoopathi et al., 2010b). SPARC-induced ER stress also leads to autophagy-mediated apoptosis (Sailaja et al., 2013). Moreover, SPARC deficiency results in decreased oxidative stress, autophagy, and superoxide-induced apoptosis (Aseer et al., 2017). Furthermore, SPARC inhibited the expression of mir-let-7f-1 by which directly inhibits high mobility group box 1 (HMGB1), a key regulator of autophagy (Pannuru et al., 2014). These findings suggested that SPARC may participate in the regulation of neuron damage in nervous system diseases by inducing autophagy.

In the CNS, glutamate-induced oxidative nerve injury is related to many neurodegeneration diseases, including epilepsy, AD, and ischemia (Thornton et al., 2017; Fricker et al., 2018). Some studies have shown that glutamate plays a critical role in nerve injury. Interestingly, two forms of glutamate-induced nerve toxicity have been recognized: N-methyl-D-aspartate (NMDA) receptor-activated excitotoxicity and non-receptor-mediated oxidative toxicity (Prentice et al., 2015). High concentrations of glutamate cause oxidative glutamate damage (Jia et al., 2013; Mao et al., 2016). Compelling evidence shows that high extracellular levels of glutamate promote epileptic seizures in temporal lobe epilepsy (TLE; Albrecht and Zieliñska, 2017). Moreover, glutamate-mediated neuroexcitatory toxicity is one of the main causes of neuronal death during seizures (Ambrogini et al., 2019). Also, autophagy activation is reported in the glutamate-induced neuronal injury model. Although SPARC has not been shown to be involved in glutamate-induced brain neuronal cell damage, the current study aimed to investigate whether it participates in glutamate-induced neuronal damage via induced autophagy pathway.

## Materials and Methods

### Reagents and Antibodies

The following reagents were used in this study: 3-methyladenine (3-MA) (HY-19312) and chloroquine (CQ) diphosphate salt (C6628, MedChemExpress, United States); glutamate (G8415), 3-(4,5-dimethylthiazol-2-yl)-2,5-diphenyltetrazolium bromide (MTT) (M5655), and dimethyl sulfoxide (DMSO) (D2650) (Sigma–Aldrich, United States). 3-MA, MTT, and CQ diphosphate salt were solubilized in phosphate-buffered saline (PBS). The following antibodies were used in this study: SPARC (D10F10), Beclin1 (3738), p62 (39749), β-actin (58169), LC3 (2775), LAMP1 (15665), horseradish peroxidase-conjugated anti-rabbit secondary antibody (93702), and horseradish peroxidase-conjugated anti-mouse secondary antibody (58802) (Cell Signaling Technology, United States). In immunofluorescence, goat anti-rabbit Alexa Fluor 488 and goat anti-mouse Alexa Fluor 594 antibodies were obtained from Thermo Fisher Scientific, United States.

### Cell Culture and Drug Treatments

HT22 mouse hippocampal neuronal cell line was purchased from the Institute of Biochemistry, and Cell Biology of the Chinese Academy of Sciences (Shanghai, China) and cultured in Dulbecco’s modified Eagle’s medium (DMEM; Gibco-BRL, United States) supplemented with 10% fetal bovine serum (FBS) and penicillin–streptomycin (100 U/mL, Gibco-BRL) in humidified air at 37°C with 5% CO_2_. The HT22 cells were inoculated in either a six-well plate or a 96-well plate the day before the experiment.

### Western Blot Assay

After drug treatments, the medium was removed, HT22 cells were rinsed with PBS and lysed with RIPA buffer containing phosphorylase inhibitors and protease inhibitors. The protein concentration of the lysate obtained by centrifugation at 15,000 rpm for 10 min at 4°C was determined by BCA assay kit (Beyotime, China). The separation glue with a concentration of 8–12% and the concentrated glue with a concentration of 5% were prepared. An equivalent of total protein was resolved on SDS-PAGE and transferred to the polyvinylidene fluoride (PVDF) membrane. Then, the membrane was blocked with 5% fat-free milk, which dissolved in TBS-T solution at room temperature for 1.5 h and probed with the following primary antibodies overnight at 4°C: rabbit anti-SPARC (1:1,000), rabbit anti-Beclin1 (1:1,000), rabbit anti-P62 (1:1,000), and mouse anti-β-actin (1:2,000). Subsequently, the membranes were incubated with the secondary antibodies (1:5,000) at room temperature for 2 h, followed by immunodetection using an enhanced chemiluminescence kit (Beyotime, China). The intensities of the protein bands were quantified using QuantityOne software (Bio-Rad, CA, United States).

### Immunofluorescence

An equivalent of 5 × 10^3^ HT22 cells/well were plated on glass coverslips in 24-well plates the day before the experiment. Then, cells were treated with glutamate for 24 h at 37°C with 5% CO_2_, fixed with 4% paraformaldehyde for 30 min, permeabilized with 0.4% Triton X-100, blocked with 5% FBS, before incubation with primary antibody overnight at 4°C. Subsequently, the cells were incubated at room temperature with fluorescent secondary antibodies for 2 h, followed by nucleus staining with diisopropylaniline (DAPI; Beyotime, China) for 8–10 min. Finally, the immunostained cells were examined under a confocal laser scanning microscope (Carl Zeiss, Oberkochen, Germany).

### Cell Viability Assay

The cell viability of HT22 cells was detected by MTT. HT22 cells (5000/100 μL) were plated in 96-well plates and treated with drugs for 24 h at 37°C with 5% CO_2_ before the experiment. Then, 10 μL MTT (0.5 mg/mL) reagent was added to each well and incubated at 37°C for 2–4 following which, the culture medium was removed and 150 μL DMSO was added to stop the reaction. Finally, the absorbance was measured at 490/570 nm using a micrometer (Tecan Group, Mannedorf, Switzerland). The cell viability was represented as the percentage of control cells.

### Trypan Blue Dye Exclusion Assay

After treatment with drugs, HT22 cells were stained with Trypan Blue Staining Cell Viability Assay (Beyotime, China). Firstly, HT22 cells were digested with 0.25% trypsin (containing EDTA) and collected by centrifugation at 1,500 rpm for 3 min. The cell pellet was resuspended and mixed with an equivalent volume of Trypan blue for 3 min to facilitate the staining of the cells. The dead cells stained blue, and the live cells were colorless and transparent. The number of blue cells and the total number of cells were counted using a hemocytometer. Mortality rate (%) = total number of dead cells/(total number of living cells + total number of dead cells) × 100%.

### Cell Transfection

First, the gene corporation (Tsingke, China) synthesized the target segments (*SPARC*-F: 5′-CCGGGAAGGTATGCA GCAATGACAACTCGAGTTGTCATTGCTGCATACCTTCTT TTTG-3′, *SPARC*-R: 5′-AATTCAAAAAGAAGGTATGCAGC AATGACAACTCGAGTTGTCATTGCTGCATACCTTC-3) (Tsingke, China).

Then, sh-SPARC plasmids were constructed. SPARC-overexpressing plasmids were purchased from OriGene China (Beijing, China). Next, these plasmids were transfected in 293 cells according to the protocol for lentivirus transfection and the packaging of the retrovirus. The virus-containing supernatant was collected after 24 h post-transfection by centrifugation at 3,000 rpm for 20 min to remove cell precipitates. The virus venom was added to HT22 cells cultured in six-well plates with 3 μL polybrene and incubated for 48 h. The expression of SPARC was determined by Western blot analysis.

### Statistical Analysis

GraphPad Prism 6.0 and SPSS 20.0 software were used for statistical analysis. The data were obtained from at least three independent experiments and expressed as mean ± SD. The statistical differences between the groups were analyzed and compared using an unpaired Student’s *t*-test or one-way analysis of variance (ANOVA), followed by a Bonferroni *post hoc* test. *P* = 0.05 and *P* < 0.05 indicated statistical significance.

## Results

### Glutamate Induced HT22 Hippocampus Nerve Cell Injury

**FIGURE 1:**
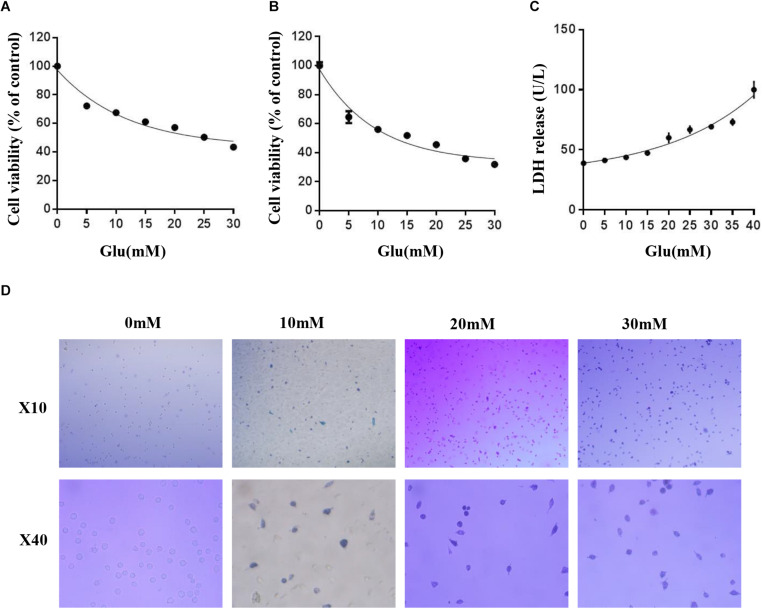
Glutamate induced HT22 hippocampus nerve cell injury. **(A)** Cell viability of HT22 cells was analyzed by MTT method at different concentrations of glutamate (0–30 mM). **(B)** Cell viability of HT22 cells was also analyzed by CCK8 assay at different concentrations of glutamate (0–30 mM). **(C)** The cell injury due to glutamate insults in HT22 cells was confirmed by LDH release assay. **(D)** HT22 cells treated with different concentrations of glutamate were stained using Trypan blue viability assay (*, *P* < 0.05; **, *P* < 0.01; ***, *P* < 0.001).

Some studies have shown that glutamate induces neuroexcitatory toxicity and causes damage to HT22 hippocampal nerve cells. Hence, to explore the optimal damage concentration of glutamate, we treated HT22 hippocampal nerve cells with various concentrations of glutamate at 37°C under 5% CO_2_ for 24 h. Then, we used the MTT method, CCK8 assay, and lactate dehydrogenase (LDH) release assay kit to determine the cell activity and LDH content in the culture medium. The cell viability was significantly decreased in a dose-dependent manner after glutamate treatment for 24 h (Figures 1A,B). When the cell activity was 50–60%, the concentration of glutamate was approximately 10 mM. The LDH release assay also confirmed this phenomenon (Figure 1C). Finally, HT22 cells treated with different concentrations of glutamate were stained using the Trypan Blue Staining Cell Viability Assay Kit. The live HT22 cells were colorless and transparent, and the dead HT22 cells were stained blue. The blue HT22 cells were increased significantly under glutamate stimulation (Figure 1D), suggesting that glutamate causes nerve cell damage in HT22 hippocampus nerve cells.

### Expression of SPARC Increased in HT22 Glutamate-Induced HT22 Cell Injury and SPARC Knockdown Reduced Glutamate-Induced HT22 Cell Injury

**FIGURE 2:**
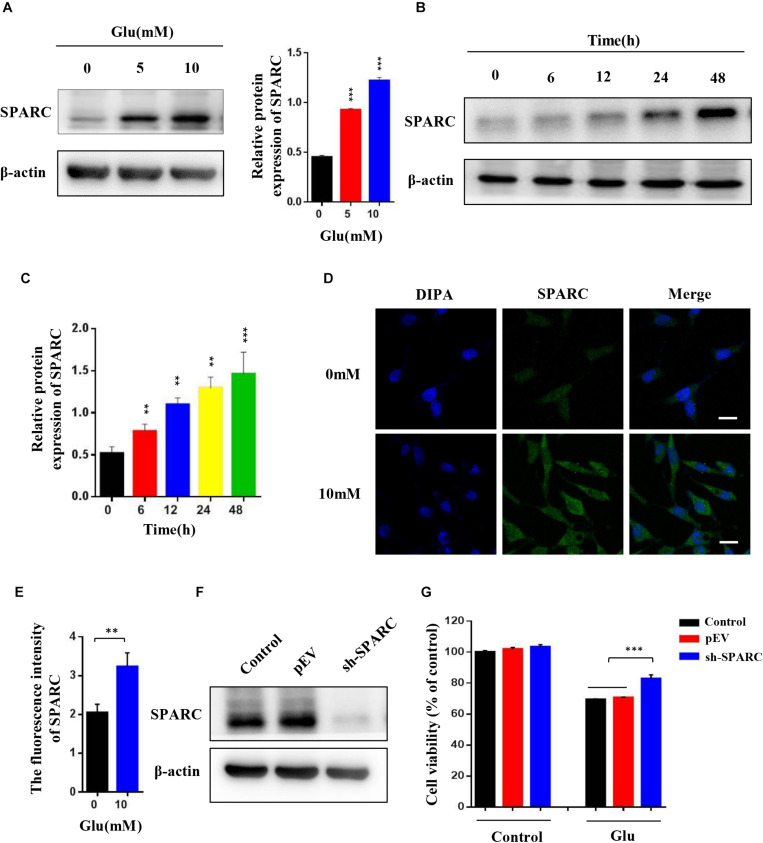
Expression of SPARC increased in HT22 glutamate-induced HT22 cell injury, and SPARC knockdown reduced glutamate-induced HT22 cell injury. **(A)** HT22 cells attached to the wall were treated with 0 mM, 5 mM, and 10 mM glutamate, respectively, for 24 h. **(B,C)** HT22 cells were treated with 10 mM glutamate for 0, 6, 12, 24, and 48 h, respectively. Western blot detected the expression levels of SPARC under glutamate stimulation. **(D,E)** The expression of SPARC was detected by immunofluorescence in glutamate-induced HT22 cell injury (Scale bar 20 μm). Quantification of the relative fluorescence intensity of SPARC in HT22 cells. **(F)** Efficiency of SPARC knockdown was detected by Western blot. **(G)** MTT method was used to detect the cell activity of SPARC knockdown in HT22 cells by stimulating with glutamate for 24 h (*P < 0.05; **P < 0.01; ***P < 0.001).

To test whether SPARC was also increased in glutamate-induced HT22 hippocampal nerve cell injury, the HT22 hippocampal nerve cells attached to the wall were treated with 0, 5, and 10 mM glutamate for 24 h and treated with 10 mM glutamate for 0, 6, 12, 24, and 48 h, respectively. The expression of SPARC was significantly increased in a dose- and time-dependent manner under glutamate stimulation (Figures 2A–C). The expression and distribution of SPARC in HT22 hippocampal nerve cell injury induced by glutamate were further detected by immunofluorescence. Herein, we treated HT22 hippocampal nerve cells attached to the wall with 10 mM glutamate for 24 h. Compared to normal HT22 cells, the expression of SPARC was increased in the glutamate-damaged model and was found in both the nucleus and cytoplasm (Figures 2D,E). The data showed that the level SPARC increased in HT22 hippocampal nerve cell injury induced by glutamate, suggesting that SPARC may be related to nerve cell injury. To further explore the role of SPARC in glutamate-induced nerve damage, we knocked down SPARC in HT22 cells, respectively. First, the knockdown efficiency of SPARC was detected by Western blot (Figure 2F). In addition, we used MTT method to detect the cell activity of SPARC knockdown HT22 cells by glutamate stimulation for 24 h. Compared to normal HT22 cells stimulated with glutamate, the cell activity was increased in the SPARC knockdown HT22 cells stimulated with glutamate for 24 h (Figure 2G). The data showed that SPARC knockdown reduced glutamate-induced HT22 nerve cell injury. Thus, SPARC may play a major role in glutamate-induced HT22 hippocampal nerve cell injury.

### Autophagy Was Activated in Glutamate-Induced HT22 Hippocampal Nerve Cell Injury

**FIGURE 3:**
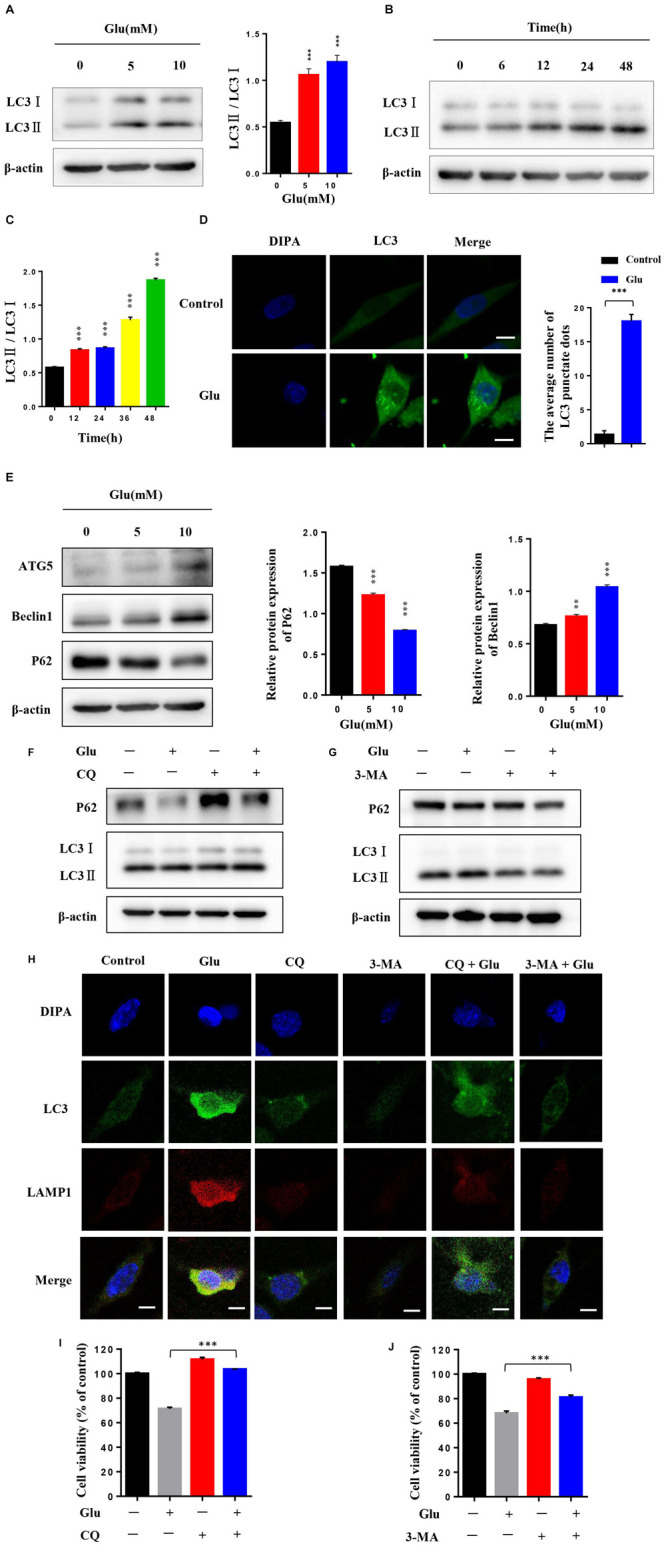
Autophagy was activated in glutamate-induced HT22 hippocampal nerve cell injury. **(A)** HT22 cells attached to the wall were treated with 0, 5, and 10 mM glutamate, respectively, for 24 h. **(B,C)** HT22 cells were treated with 10 mM glutamate for 0, 6, 12, 24, and 48 h, respectively. Western blot detected the expression levels of LC3II. **(D)** Expression level of LC3 was further detected by immunofluorescence (Scale bars 10 μm). **(E)** Western blot assay of the expression of autophagy protein in HT22 cells treated with glutamate. **(F,G)** Expression of LC3II/LC3I and P62 was detected by Western blot in HT22 cells with or without autophagy inhibitor (CQ and 3-MA). **(H)** Representative confocal images for LC3 (green), LAMP1 (red), and nuclei (blue) of HT22 cells treated with autophagy inhibitor (CQ or 3-MA) and glutamate (10 mM) for 24 h (Scale bars 10 μm). **(I,J)** HT22 cells treated with autophagy inhibitor (CQ or 3-MA) were stimulated by glutamate. Then, cell viability was determined (*P < 0.05; **P < 0.01; ***P < 0.001).

Current studies have shown that autophagy is associated with a variety of nerve injuries in CNS diseases. Some studies have also shown that autophagy is involved in glutamate-induced nerve damage. To detect the autophagy levels in HT22 hippocampal nerve cell injury induced by glutamate, the nerve cells attached to the wall were treated with 0, 5, and 10 mM glutamate for 24 h and independently treated with 10 mM glutamate for 0, 6, 12, 24, and 48 h, respectively. Also, the level of autophagy was significantly increased in a dose- and time-dependent manner under glutamate stimulation (Figures 3A–C). In addition, the expression levels of LC3II increased in a concentration-dependent and time-dependent manner. Moreover, the expression level of LC3 was further detected by immunofluorescence. After 24 h post-HT22 hippocampal nerve cell stimulation by 10 mM glutamate, the average number of LC3 spots in the glutamate damage model was increased significantly compared to the normal HT22 cells (Figure 3D). In addition, the expression of beclin1 increased and that of p62 decreased after HT22 hippocampal nerve cells were treated with 0, 5, and 10 mM glutamate for 24 h, respectively (Figure 3E). These findings suggested that autophagy is activated after glutamate stimulation. We also examined whether autophagy flow is unobstructed. Thus, we used glutamate combined with autophagy inhibitors (CQ and 3-MA) to reflect the autophagy flow by detecting the expression of LC3 and P62. Compared to HT22 cells with glutamate stimulation, the expression of LC3II was increased, and that of p62 was reduced in HT22 cells stimulated with glutamate and CQ for 24 h (Figure 3F). Furthermore, compared to HT22 cells stimulated with glutamate, the expression of LC3II and P62 was increased in HT22 cells stimulated simultaneously with glutamate and 3-MA for 24 h (Figure 3G). Finally, the co-localization of LC3 and LAMP1 was detected in nerve cells. In HT22 cells treated with glutamate, LC3 and LAMP1 showed good co-localization; however, in HT22 cells simultaneously stimulated with glutamate and CQ for 24 h, LC3 and LAMP1 showed low co-localization (Figure 3H). The data showed that autophagy flow was unobstructed after glutamate stimulation. In order to investigate the effect of autophagy on glutamate-induced nerve injury, we conducted an MTT assay on glutamine-treated cells in combination with autophagy inhibitors (CQ and 3-MA). The autophagy inhibitors increase the activity of glutamate-treated HT22 cells, suggesting that autophagy promotes glutamate-induced HT22 cell death (Figures 3I,J). These findings suggested that autophagy is activated in HT22 hippocampal nerve cell injury induced by glutamate, and autophagy may be involved in nerve cell injury.

### SPARC Regulated Autophagy in Glutamate-Induced HT22 Hippocampal Nerve Cell Injury

**FIGURE 4:**
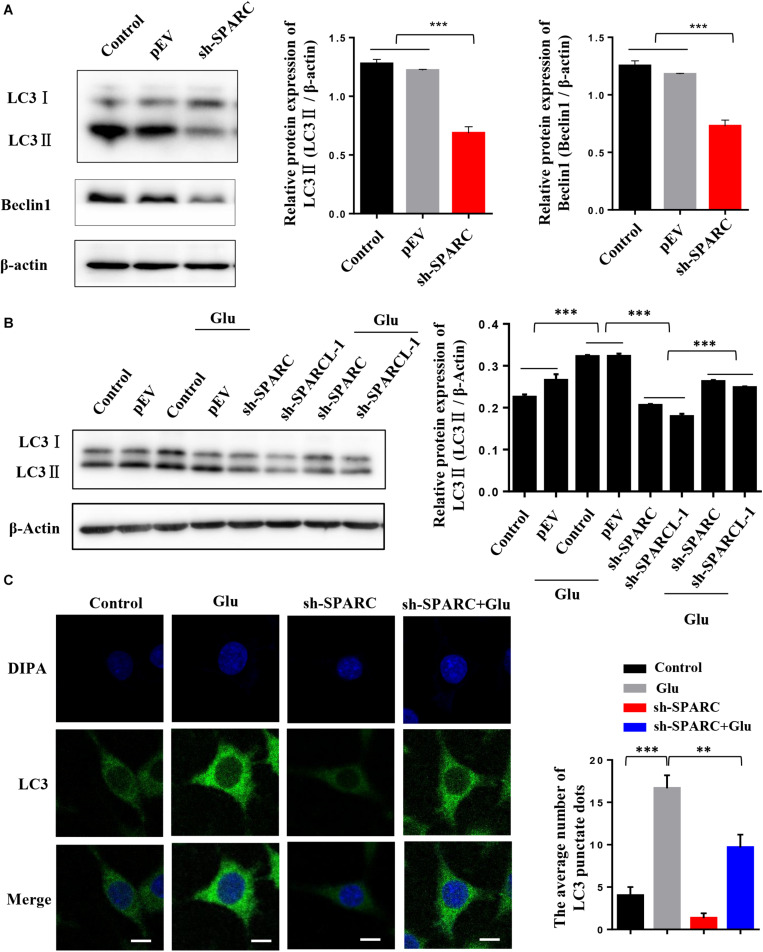
SPARC regulated autophagy in glutamate-induced HT22 hippocampal nerve cell injury. **(A)** Expression level of LC3 and Beclin1 was detected by Western blot in SPARC-knockdown HT22 cells. Then, SPARC-knockdown HT22 cells were stimulated by glutamate (10 mM) for 24 h. **(B)** Expression level of LC3 was detected by Western blot. **(C)** Expression level of LC3 was further detected by immunofluorescence (Scale bars 10 μm) (*P < 0.05; **P < 0.01; ***P < 0.001).

To further verify the regulatory effect of SPARC on autophagy, we measured the level of autophagy in SPARC knockdown HT22 cells. The data showed that the expression of LC3II and Beclin1 decreased after SPARC knockdown (Figure 4A). We also overexpressed SPARC to detect autophagy in HT22 cells (Supplementary Figure 1A). In SPARC-overexpressing HT22 cells, the results of Western blot showed that the expression of LC3II and Beclin1 was increased, and that of P62 was decreased (Supplementary Figures 1B,C). Immunofluorescence showed that autophagy was increased in SPARC-overexpressed HT22 cells (Supplementary Figure 1D). These findings suggested that SPARC regulates autophagy. SPARC-knockdown HT22 cells were treated with glutamate for 24 h. The level of autophagy marker LC3 was detected by Western blot and immunofluorescence. Compared to normal cells treated with glutamate, SPARC knockdown significantly reduced autophagy levels in glutamate-induced nerve injury (Figures 4B,C). These findings suggested that SPARC can reduce glutamate-induced HT22 hippocampal nerve cell injury by the regulation autophagy.

## Discussion

Secreted protein acidic and rich in cysteine has been linked to the activity of growth factors, cytokines, and ECM in tissue development and repair via regulation of angiogenesis and cellular adhesion, migration, and proliferation (Bradshaw, 2012). However, the exact function of SPARC on the regulation of critical cellular activity in nervous system disease is yet to be elucidated. The main findings of the current study exhibited that SPARC was increased in glutamate-induced neuron damage and regulated autophagy in HT22 hippocampal nerve cells. Moreover, low expression of SPARC reduced glutamate-induced HT22 hippocampal nerve cell damage. These findings indicated that SPARC knockdown reduces glutamate-induced HT22 hippocampal nerve cell damage via autophagy.

In the CNS, glutamate is an excitatory neurotransmitter associated with the regulation of cognition and synaptic plasticity in the development of CNS (Danbolt et al., 2016). However, high levels of glutamate cause significant oxidative glutamate toxicity and nerve cell injury, which are closely related to the pathogenesis of various CNS diseases, such as ischemic stroke, epilepsy, and AD (Mao et al., 2015; Albrecht and Zieliñska, 2017; Yang et al., 2019). Glutamate-mediated excitatory toxicity, neuroinflammation, and oxidative stress are standard features in neurodegenerative diseases. Furthermore, the neurobiological feature of epilepsy is involved in this pathogenic triad, resulting in nerve cell death and subsequently enhancing the sensitivity to neuronal synchronization and network changes in epilepsy (Ambrogini et al., 2019). Persistent high levels of extracellular glutamate might be the main cause of excitotoxicity in epileptic seizures. Therefore, the glutamate-induced nerve injury model was used to explore nerve injury. MTT, CCK8, and LDH release assays and Trypan blue viability assay confirmed that glutamate-induced hippocampal nerve cell injury *in vitro*.

The SPARC family of proteins is mainly involved in modulating cell interaction with the ECM. Notably, the function of SPARC family members, including SPARC-L, remains unclear in CNS, although these proteins are highly expressed in the brain (Chen et al., 2020). It has also been confirmed that SPARC is involved in CNS injury and the recovery of nerve function after injury via the modulated response of microglial cells in gray and white matter (Lloyd-Burton et al., 2013). However, among the SPARC family proteins, the level of SPARCL-1 was increased and localized to excitatory synapses following status epilepticus (SE) in the rat lithium-pilocarpine seizure model. The upregulated expression of SPARCL-1 may be involved in synaptic plasticity of neuronal degeneration and the stress of nerve damage caused by epileptic seizures in brain tissue (Lively and Brown, 2008a,b). Additionally, the expression of SPARCL-1 is significantly increased in adult rats after transient ischemic stroke and in the rat striatum after acute injury (Lively et al., 2011; Lively and Schlichter, 2012). SPARC is also shown to augment the apoptotic cascade and is associated with damage in a variety of cells (Bhoopathi et al., 2010b; Rahman et al., 2011; Chern et al., 2019). Although SPARC and SPARCL-1 have been explored in animal brain tissue, nerve injury expression and regulatory mechanisms are not fully understood. SPARCL-1 is highly homologous to SPARC, and hence, we only explored the role of SPARC in nerve injury. However, the current study confirmed that the expression of SPARC was increased in glutamate damage models. Subsequently, SPARC was knocked down in HT22 hippocampal nerve cells treated with glutamate. Then, the cell activity was determined, which confirmed that SPARC knockdown reduced the glutamate-induced nerve injury. This study demonstrated that SPARC was involved in glutamate-induced nerve injury.

Current studies have also shown that the autophagy pathway might play a major role in the nervous system disease (Moloudizargari et al., 2017; Wang et al., 2018). Our study confirmed that autophagy is involved in glutamate-induced nerve injury. Under glutamic acid stimulation, LC3II/LC3I increases, and consequently, autophagy is activated. However, we used autophagy inhibitors (CQ and 3-MA) to reduce the glutamate-induced nerve injury. Some studies have shown that SPARC regulates the occurrence of autophagy under different pathological conditions. Also, SPARC activates autophagy-mediated apoptosis in medulloblastoma and neuroblastoma primitive neuroectodermal tumor (PNET) cells via different pathways (Bhoopathi et al., 2010b; Pannuru et al., 2014). In addition, SPARC overexpression also leads to autophagy-mediated apoptosis in neuroblastoma by triggering ER stress and unfolded protein response (UPR; Sailaja et al., 2013). Notably, apoptosis can be enhanced by SPARC. Nonetheless, SPARC not only activates autophagy but is also involved in apoptosis, albeit the exact regulatory mechanism is not yet understood. However, as a potential chemical sensitizer, SPARC has attracted increasing attention due to its ability to enhance apoptosis cascade and autophagy (Rahman et al., 2011; Notaro et al., 2016). Despite autophagy being profusely described in the context of tumor tissue, these studies suggested that SAPRC may affect nerve injury by regulating autophagy in neurological diseases. The present study also demonstrated that autophagy was activated in SPARC-overexpressed HT22 cells. Compared to normal HT22 cells treated with glutamate, autophagy decreased in SPARC-knockdown HT22 cells treated with glutamate. These findings confirmed that SPARC regulated autophagy and affected glutamate-induced hippocampal nerve injury.

Although we explored the effect of SPARC on nerve injury by regulating autophagy, other factors affecting nerve injury cannot be ruled out. In addition to directly regulating nerve death, SPARC is also involved in regulating synapses and nerve factors to affect nerve function. In the developing brain, SPARCL-1 directly enhances the formation of an excitatory synapse, and the synaptic generation is specifically inhibited by SPARC (Blakely et al., 2015). Moreover, SPARCL-1 might enhance the connectivity of synapse and reduce the incidence of neurodegeneration in the brain by reducing the formation of NMDA receptors, augmenting the numbers of the synapse, and increasing the branches of dendrites (Kucukdereli et al., 2011). Additionally, SPARCL-1 directly increases the recruitment of the NMDA receptor and the formation of synapse (Jones et al., 2011; Gan and Südhof, 2019). However, SPARC might be involved in the regulation of AMPARs and GluRs in the damage and repair of CNS (Jones et al., 2011, 2018). The present study also found that SPARC directly interacts with NGFβ *in vitro*, thereby regulating NGFβ functions (Okura et al., 2019). Remarkably, NMDARs or GluRs is a receptor that interacts with glutamate, and their activation causes excitatory toxicity of nerve cells. Therefore, the increase in the number of NMDARs or GluRs may be one of the causes of glutamate-induced nerve cell death, which was substantiated in our future studies on SPARC and SPARCL-1. In addition, SPARC regulates angiogenesis and inflammation (Llera et al., 2010; Rivera et al., 2011). These findings suggested that SPARC may regulate nerve damage in the brain by regulating angiogenesis and inflammatory responses, thereby contributing to the research, prevention, and treatment of cerebrovascular diseases, AD, and epilepsy. The *in vitro* and *in vivo* data showed the expression of SPARC during the calcification process and suggested its potential role as a procalcifying factor (Ciceri et al., 2016). However, SPARC as a procalcifying factor may affect the distribution of calcium ions in nerve cells, resulting in nerve cell damage. These data suggested that SPARC may be involved in the regulation of nerve injury in CNS diseases. Also, SPARC is also closely related to diabetes, glucose metabolism, and fat metabolism (Kos and Wilding, 2010). Therefore, the study on the functions of SPARC may reduce the damage caused by ischemia, hypoxia, hyperglycemia, and hyperlipidemia to brain tissue in order to promote novel research on the occurrence and development of neurological diseases.

Currently, the correlation between neurological diseases, such as epilepsy, PD, AD, and ischemia, and SPARC needs to be investigated further. Moreover, in the SPARC family, the role of SPARCL-1 as a homologous protein of SPARC in neuron damage has not been explored, and whether molecules containing the same region activate autophagy is not yet elaborated and will be explored in the future.

## Conclusion

In summary, this study found that SPARC plays a major role in nerve injury, especially epilepsy. SPARC regulates autophagy in glutamate-induced HT22 hippocampus nerve cell injury. On the other hand, low SPARC inhibits the autophagy pathway and reduces glutamate-induced HT22 hippocampus nerve cell injury. Therefore, regulating the expression of SPARC may be the key to the prevention and control of neurological diseases, such as epilepsy, PD, AD, and ischemia.

## Data Availability Statement

The original contributions presented in the study are included in the article/Supplementary Material, further inquiries can be directed to the corresponding author/s.

## Author Contributions

SC participated in experimental studies. SC, QZ, and YC searched and sorted the references and participated in drafting the manuscript. SC and XK were involved in the literature search. QL and QZ coordinated and supervised the study, provided research direction, designed the research strategy, and modified the final draft. All authors have carefully read and confirmed the final manuscript.

## Conflict of Interest

The authors declare that the research was conducted in the absence of any commercial or financial relationships that could be construed as a potential conflict of interest.

## Supplementary Material

Supplementary Figure 1 **(A)** Western blot showed the overexpression of SPARC in HT22 cells. **(B)** Expression level of LC3 was detected by Western blot in SPARC-overexpressed HT22 cells. **(C)** Western blot assay of expression of autophagy protein in SPARC-overexpressed HT22 cells. **(D)** Expression level of LC3 was further detected by immunofluorescence in SPARC-overexpressed HT22 cells (Scale bars 10 μm) (^∗^*P* < 0.05; ^∗∗^*P* < 0.01; ^∗∗∗^*P* < 0.001).
