# Supplementary figures and images for: SPARC Knockdown Reduces Glutamate-Induced HT22 Hippocampal Nerve Cell Damage by Regulating Autophagy
Source: Front Neurosci. 2021 Jan 26;14:581441. doi: 10.3389/fnins.2020.581441 (PMC7874057; doi:10.3389/fnins.2020.581441)

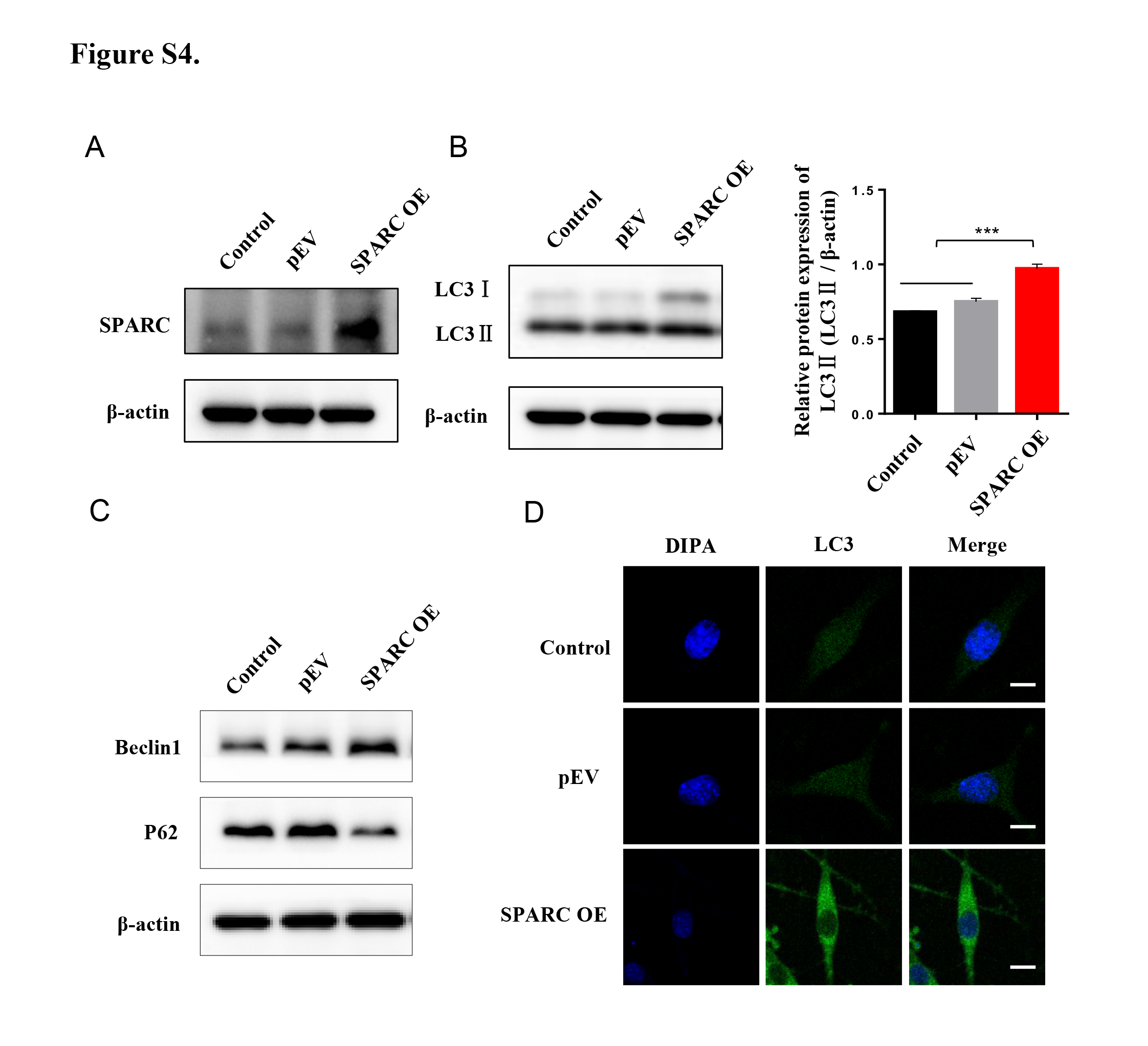

Supplement: Supplementary Figure 1 — (A) Western blot showed the overexpression of SPARC in HT22 cells. (B) Expression level of LC3 was detected by Western blot in SPARC-overexpressed HT22 cells. (C) Western blot assay of expression of autophagy protein in SPARC-overexpressed HT22 cells. (D) Expression level of LC3 was further detected by immunofluorescence in SPARC-overexpressed HT22 cells (Scale bars 10 μm) (∗P < 0.05; ∗∗P < 0.01; ∗∗∗P < 0.001). [file Image_1.TIF]
